# TAB2 Promotes Immune Escape and Chemoresistance Through NF‐κB Pathway Activation in Cervical Cancer

**DOI:** 10.1111/jcmm.70522

**Published:** 2025-03-25

**Authors:** Man Wu, Yingying Zhang, Xuanhui Wang, Yijia Zhou

**Affiliations:** ^1^ Key Laboratory for Reproductive Medicine of Guangdong Province Third Affiliated Hospital of Guangzhou Medical University Guangzhou China; ^2^ Department of Obstetrics and Gynecology, Center for Reproductive Medicine, Guangdong Provincial Key Laboratory of Major Obstetric Diseases The Third Affiliated Hospital of Guangzhou Medical University Guangzhou Guangdong China; ^3^ Department of Obstetrics and Gynecology The First Affiliated Hospital, Sun Yat‐sen University Guangzhou China; ^4^ Guangdong Provincial Clinical Research Center for Obstetrical and Gynecological Diseases Guangzhou Guangdong China

**Keywords:** cervical cancer, chemoresistance, immune escape, NF‐κB pathway, TAB2

## Abstract

Cervical cancer (CC) remains a major health challenge with high mortality rates due to chemoresistance and immune escape. However, the underlying mechanisms remain unclear. We investigated the role of TAB2 in CC using cisplatin‐resistant and parental cell lines. Cell proliferation, migration, sphere formation and T cell‐mediated killing assays were performed. Western blot and qRT‐PCR analysed protein and mRNA expression. NF‐κB pathway involvement was examined using the BAY 11–7082 inhibitor. TAB2 expression was significantly elevated in cisplatin‐resistant CC cells. TAB2 overexpression promoted chemoresistance and immune escape through NF‐κB pathway activation. Conversely, TAB2 knockdown or NF‐κB inhibition sensitised resistant cells to cisplatin and enhanced T cell‐mediated killing. The resistant phenotype could be rescued by restoring PD‐L1 expression. Our findings reveal TAB2 as a critical regulator of both chemoresistance and immune escape in CC through NF‐κB pathway activation. This suggests TAB2 as a potential therapeutic target for overcoming treatment resistance in CC.

**Abbreviations**
ALDH: Aldehyde Dehydrogenase
BAY 11–7082: NF‐κB Inhibitor
BMI1: B cell‐specific Moloney murine leukaemia virus integration site 1
CC: cervical cancer
CCK‐8: Cell Counting Kit‐8
CDDP: Cisplatin
CO_2_
: carbon dioxide
CSCs: cancer stem cells
DEAB: Diethylaminobenzaldehyde
DED: dry eye disease
DMEM: Dulbecco's Modified Eagle's Medium
DNA: Deoxyribonucleic Acid
ECL: Enhanced Chemiluminescence
EGF: Epidermal Growth Factor
EMT: Epithelial‐Mesenchymal Transition
FBS: foetal bovine serum
FITC/PI: Fluorescein Isothiocyanate/Propidium Iodide
Gal‐3: Galectin‐3
GAPDH: Glyceraldehyde 3‐phosphate Dehydrogenase
HPV: human papillomavirus
hucMSC‐EVs: Human Umbilical Cord Mesenchymal Stem Cell‐derived Extracellular Vesicles
IC50: Half Maximal Inhibitory Concentration
IKB: Inhibitor of Nuclear Factor Kappa B
IKK: IκB Kinase
IRAK1: Interleukin‐1 Receptor‐Associated Kinase 1
MAPK: Mitogen‐Activated Protein Kinase
MEM: minimum essential medium
miRNA: MicroRNA
MS751‐R: Cisplatin‐resistant MS751 Cell Line
NF‐κB: Nuclear Factor Kappa B
PBS: Phosphate‐Buffered Saline
PD‐L1: Programmed Death‐Ligand 1
PVDF: Polyvinylidene Fluoride
qRT‐PCR: Quantitative Real‐Time Polymerase Chain Reaction
RI: Resistance Index
RIPA: Radioimmunoprecipitation Assay
SDS‐PAGE: Sodium Dodecyl Sulphate Polyacrylamide Gel Electrophoresis
Siha‐R: Cisplatin‐resistant Siha Cell Line
siRNA: Small Interfering RNA
SOX2: SRY‐box Transcription Factor 2
TAB2: TAK1‐binding Protein 2
TAK1: Transforming Growth Factor Beta‐activated Kinase 1
TBST: Tris‐buffered Saline with Tween 20
TNFR1: Tumour Necrosis Factor Receptor 1
TRAF6: TNF Receptor‐Associated Factor 6
USP25: Ubiquitin‐Specific Peptidase 25

## 1 Introduction

Cervical cancer (CC) is the fourth most prevalent malignancy among women worldwide, and the second most common in developing countries [1]. Despite the effectiveness of human papillomavirus (HPV) vaccination in preventing CC, its suboptimal prevalence, particularly in developing countries, coupled with the poor efficacy and prognosis of advanced CC, presents significant challenges [2]. Chemoresistance and immune evasion remain major obstacles in the treatment of CC, making a deeper understanding of the molecular mechanisms driving these challenges crucial [3, 4]. The nuclear factor‐κB (NF‐κB) signalling pathway is a critical pathway involved in the regulation of cell proliferation, invasion, inflammatory reactions and tumour development. As a central component of the cell signalling transduction network, NF‐κB plays diverse roles in CC development, with its activity varying during different stages of HPV infection [5]. Specifically, abnormal activation of NF‐κB is closely related to the progression of CC, promoting tumour formation and development through various mechanisms, including the regulation of the tumour microenvironment and the acquisition of cancer hallmarks. Moreover, NF‐κB has been implicated in chemoresistance and immune regulation in CC, making it a potential prognostic biomarker and therapeutic target. Therefore, a thorough investigation of the role and mechanisms of the NF‐κB signalling pathway in CC is essential for developing novel therapeutic strategies and overcoming chemoresistance and immune evasion [6, 7].

Transforming growth factor‐β‐activated kinase 1 (TAK1)‐binding protein 2 (TAB2) is an adaptor protein that transmits signals from tumour necrosis factor receptor 1 (TNFR1) and other receptors to the TAK1 signalling complex by binding to lysine‐63‐linked polyubiquitin chains. TAB2 plays a crucial regulatory role in the TAK1 signalling pathway, functioning as both an activator and an inhibitor of TAK1, depending on the cell type or cellular context [8, 9]. Aberrant expression and function of TAB2 have been implicated in the development and progression of various cancers. Our previous study has shown that TAB2 is significantly upregulated in CC stem cells, and that its expression level is negatively correlated with the overall survival rate of patients with CC. Furthermore, TAB2 has been found to promote the development and progression of CC by regulating the properties of cancer stem cells (CSCs) [10]. However, despite these findings suggesting an important role for TAB2 in certain cancers, the specific role and mechanisms of TAB2 in CC, and how it interacts with other signalling pathways, such as NF‐κB, remain poorly understood. Therefore, in‐depth research into the mechanisms of TAB2 in CC is critical for developing new therapeutic strategies.

In this study, we investigated the molecular mechanisms by which TAB2 promotes CC progression through NF‐κB pathway activation. Using CC cell lines and established cisplatin‐resistant variants, we demonstrated that TAB2 regulates multiple aspects of cancer cell behaviour including stemness properties, cell growth, migration and immune escape. Importantly, we revealed that TAB2 modulates PD‐L1 expression through NF‐κB signalling, contributing to immune evasion. Furthermore, we uncovered TAB2's crucial role in mediating chemoresistance and identified the TAB2‐NF‐κB axis as a potential therapeutic target. Our findings provide novel insights into the molecular mechanisms underlying CC progression and treatment resistance, suggesting that targeting TAB2 or its downstream pathway components might represent a promising strategy for overcoming both chemoresistance and immune escape in CC therapy.

## 2 Materials and Methods

### 2.1 Cell Lines and Culture

The human CC cell lines (Siha and MS751) and normal cervical epithelial cell line H8 were purchased from the Type Culture Collection of the Chinese Academy of Sciences (Shanghai, China). Siha cells were maintained in Dulbecco's Modified Eagle's Medium (DMEM, Gibco, 11,885–076) supplemented with 10% foetal bovine serum (FBS, Gibco, 10,270–106) and 1% penicillin–streptomycin (Gibco, 15,140,122). MS751 cells were cultured in Minimum Essential Medium (MEM, Gibco, 11,095,080) supplemented with 10% FBS and 1% non‐essential amino acids (Gibco, 11,140–050). All cells were maintained at 37°C with 5% CO_2_.

To establish cisplatin‐resistant cell lines (Siha‐R and MS751‐R), parental cells were exposed to gradually increasing concentrations of cisplatin (starting from IC50 value) for 6 months. The initial concentration was 5 μM, which was increased by 5 μM every 2 weeks until reaching 50 μM. The resistant phenotype was maintained by culturing cells in medium containing 10 μM cisplatin. Resistance was confirmed by IC50 determination using CCK‐8 assay. For BAY 11–7082 treatment, cells were treated with 5 μM BAY 11–7082 (Selleck Chemicals, S2913) for 24 h before subsequent experiments.

### 2.2 Plasmids and Cell Transfection

TAB2 siRNAs were designed and synthesised by GenePharma (Shanghai, China). For TAB2 overexpression, the full‐length human TAB2 cDNA was cloned into the pcDNA3.1 vector (Invitrogen) to generate vector‐TAB2. Similarly, human PD‐L1 cDNA was cloned into the pcDNA3.1 vector to construct vector‐PD‐L1. All constructs were verified by DNA sequencing. Cell transfection was performed using Lipofectamine 3000 reagent (Invitrogen, L3000015) according to the manufacturer's protocol. Briefly, cells were seeded in plates and grown to 60%–70% confluence. For siRNA transfection, 50 nM siRNA was used. For plasmid transfection, 2 μg/mL plasmid DNA was used. The transfection mixture was prepared in Opti‐MEM medium (Gibco, 31,985,070) and added to cells for 6 h before changing to complete medium. Cells were harvested 48 h post‐transfection for subsequent experiments. Transfection efficiency was verified by Western blot or qRT‐PCR analysis.

### 2.3 Western Blot Analysis

Cellular proteins were extracted using ice‐cold RIPA lysis buffer supplemented with protease and phosphatase inhibitor cocktail. Protein concentration was determined using Pierce BCA Protein Assay Kit (Thermo Fisher Scientific, 23,227). Equal amounts of protein (30 μg) were separated by 10% SDS‐PAGE and transferred onto PVDF membranes (Millipore, Germany). The membranes were blocked with 5% non‐fat milk in TBST for 1 h at room temperature and then incubated with primary antibodies overnight at 4°C. The following primary antibodies were used: anti‐TAB2 (1:1000, Proteintech, 14,410‐1‐AP‐50 μL), anti‐BMI1 (1:1000, Abcam, ab14389‐25 μg), anti‐SOX2 (1:1000, Abcam, ab97959‐100 μg), anti‐IKB (1:1000), anti‐p‐IKB (1:1000), anti‐p‐NF‐κB (1:1000), anti‐PD‐L1 (1:1000) and anti‐GAPDH (1:2000, Abcam, ab70699). After washing three times with TBST, membranes were incubated with HRP‐conjugated secondary antibodies (1:5000) for 1 h at room temperature. Protein bands were visualised using enhanced chemiluminescence (ECL) reagent and detected using the Bio‐Rad ChemiDoc imaging system. Band intensities were quantified using ImageJ software (NIH, USA) with background subtraction. All protein levels were normalised to GAPDH expression from the same sample. For phosphorylated proteins, levels were normalised to both GAPDH and their corresponding total protein levels. Quantification was performed on three independent experiments, and the mean ± SD was calculated.

### 2.4 Quantitative Real‐Time PCR

Cellular total RNA was extracted using TRIzol reagent (Invitrogen, 15,596,026) according to the manufacturer's instructions. RNA concentration and purity were measured using a NanoDrop spectrophotometer. One microgram of total RNA was reverse transcribed to cDNA using the PrimeScript RT Master Mix kit (TaKaRa, RR036A) following the standard procedure. Real‐time PCR was performed using the TB Green Premix Ex Taq II kit (TaKaRa, RR820B) on the Bio‐Rad CXF96 real‐time system. The reaction conditions were as follows: initial denaturation at 95°C for 30 s, followed by 40 cycles of 95°C for 5 s and 60°C for 30 s. GAPDH was used as the internal reference for calculating relative expression levels using the 2^−ΔΔCt^ method. All reactions were performed in triplicate, and the results were averaged.

### 2.5 Cell Proliferation and Survival Assays

Cell proliferation and viability were assessed using the Cell Counting Kit‐8 (CCK‐8, Dojindo, CCK8‐500) according to the manufacturer's instructions. For proliferation assays, cells were seeded in 96‐well plates at a density of 2 × 103 cells/well in 100 μL complete medium. At indicated time points (0, 24, 48 and 72 h), 10 μL of CCK‐8 reaction reagent was added to each well and incubated at 37°C for 1 h. The absorbance was measured at 450 nm using a microplate reader (Infinite 200 PRO). For drug sensitivity assays, cells were seeded in 96‐well plates at 5 × 103 cells/well and allowed to attach overnight. Cells were then treated with various concentrations of cisplatin (0, 50, 100 and 150 μM) for 48 h. Cell viability was determined using the CCK‐8 assay as described above. The IC50 values were calculated using GraphPad Prism 8.0 software. For T cell‐mediated killing assays, CC cells were co‐cultured with activated T cells at different ratios (T cell: cancer cell = 0:1, 5:1, 10:1) for 24 h. Cell viability was measured using the CCK‐8 assay, and the survival rate was calculated as the percentage relative to the untreated control group. All experiments were performed in triplicate and repeated at least three times independently.

### 2.6 Colony Formation Assay

Colony formation ability was evaluated by seeding cells in 6‐well plates at a density of 2 × 103 cells per well in complete medium. Cells were cultured at 37°C with 5% CO_2_ for 14 days, with medium changes every 3 days. At the end of the experiment, cells were fixed with 4% paraformaldehyde for 30 min at room temperature and stained with 0.1% crystal violet solution for 30 min. After carefully washing with PBS to remove excess stain, plates were air‐dried at room temperature. Colonies containing more than 50 cells were counted under a light microscope. Images of the colonies were captured using a digital camera, and colony numbers were quantified. Each group was assessed in triplicate, and experiments were independently repeated three times. For experiments involving drug treatment or gene manipulation, cells were treated with cisplatin or transfected with indicated constructs 24 h after seeding, and then cultured for colony formation as described above.

### 2.7 Sphere Formation Assay

The sphere formation assay was performed to evaluate cancer stem cell properties. Following previously described methods, cells were dissociated into single cells and seeded in 6‐well ultra‐low attachment plates (Corning) at a density of 3 × 103 cells/mL. Cells were cultured in stem cell medium consisting of DMEM/F12 (Gibco) supplemented with B27 (1:50, Invitrogen), 20 ng/mL EGF (PeproTech), 20 ng/mL bFGF (PeproTech) and 1% penicillin–streptomycin. Fresh stem cell medium was added every 2 days. After incubation for 1–2 weeks at 37°C with 5% CO_2_, spheres were observed and counted under a microscope. Spherical cell clusters with a diameter larger than 75 μm were considered as spheres formed by the self‐renewal of CSCs. For sphere formation efficiency calculation, the number of spheres was counted and divided by the initial number of seeded cells. For serial passage experiments, primary spheres were collected and dissociated into single cells using 0.25% trypsin–EDTA, then replated to generate secondary spheres. After six generations of sphere formation, we obtained a group of cells with greater stemness, named Siha sphere. Images were captured using an inverted microscope, and sphere formation efficiency was calculated for each experimental group. All experiments were performed in triplicate.

### 2.8 Migration Assay

Cell migration ability was assessed using Transwell chambers (Merck Millibo, MCHT06H48) with 8 μm pore size membranes. Briefly, cells were harvested and resuspended in serum‐free medium. A total of 1 × 104 cells in 200 μL serum‐free medium were seeded into the upper chamber, while the lower chamber was filled with 600 μL complete medium containing 20% FBS as a chemoattractant. After incubation at 37°C with 5% CO_2_ for 24 h, non‐migrated cells on the upper surface of the membrane were gently removed using a cotton swab. The cells that had migrated to the lower surface were fixed with 4% paraformaldehyde at 4°C for 30 min and stained with crystal violet at room temperature for 45 min. After washing with PBS, migrated cells were photographed under a light microscope from five randomly selected fields per well. The number of migrated cells was counted using ImageJ software. Each experiment was performed in triplicate and repeated three times independently. For experiments involving TAB2 overexpression or BAY 11–7082 treatment, cells were pre‐treated accordingly before performing the migration assay.

### 2.9 IC50 Determination and Establishment of Cisplatin‐Resistant Cell Lines

To establish cisplatin‐resistant cell lines, parental Siha and MS751 cells were exposed to gradually increasing concentrations of cisplatin (CDDP, Sigma‐Aldrich) for 6 months. The initial concentration was determined based on the IC50 value of parental cells. Starting from 5 μM, the cisplatin concentration was increased by 5 μM every 2 weeks until reaching 50 μM. The established resistant cell lines (Siha‐R and MS751‐R) were maintained in medium containing 10 μM cisplatin to maintain the resistant phenotype. For IC50 determination, cells were seeded in 96‐well plates at 5 × 103 cells per well and allowed to attach overnight. The next day, cells were treated with various concentrations of cisplatin (0, 50, 100 and 150 μM) for 48 h. Cell viability was assessed using the CCK‐8 assay as described above. The absorbance was measured at 450 nm using a microplate reader. The survival rate was calculated as follows: Survival rate (%) = (ODtreated−ODblank)/(ODcontrol−ODblank) × 100%. IC50 values were calculated using GraphPad Prism 8.0 software by fitting the dose–response curves. The resistance index (RI) was calculated as: RI = IC50 of resistant cells/IC50 of parental cells.

### 2.10 Flow Cytometry Analysis

ALDH activity was determined using the ALDEFLUOR fluorescent kit (STEM Cell Technology, 01700) according to the manufacturer's protocol. Briefly, cells were suspended in ALDEFLUOR assay buffer containing ALDH substrate (BAAA) at a concentration of 1 × 106 cells/mL. For each sample, a test tube containing the cell suspension with activated ALDEFLUOR substrate was prepared. As a negative control, an aliquot of the same cell suspension was treated with 5 μL of diethylaminobenzaldehyde (DEAB), a specific ALDH inhibitor. Both samples were incubated for 30 min at 37°C. After incubation, cells were centrifuged and cell pellets were resuspended in ALDEFLUOR assay buffer to analyse ALDH activity. Analysis was performed using a flow cytometer with BD FACSDiva software 6.0 (BD Biosciences, New York, NY, USA). For T cell‐mediated killing assays, CC cells were co‐cultured with activated T cells at different ratios. After 24 h of co‐culture, cells were collected and stained with Annexin V‐FITC/PI according to the manufacturer's instructions. The percentage of apoptotic cells was analysed by flow cytometry. All experiments were performed in triplicate, with gates set based on negative controls. Data analysis was performed using FlowJo software.

### 2.11 Statistical Analysis

All data are presented as mean ± standard deviation (SD) from at least three independent experiments. Statistical comparisons between two groups were performed using Student's *t*‐test. For comparisons involving more than two groups, one‐way analysis of variance (ANOVA) was used, followed by Tukey's post hoc test for multiple comparisons. Cell viability, colony formation and sphere formation assays were analysed using two‐way ANOVA followed by Bonferroni's post hoc test. The correlation between TAB2 expression and clinicopathological features was assessed using the chi‐square test. Survival analysis was performed using the Kaplan–Meier method with log‐rank test. For Western blot quantification, band intensities were measured using ImageJ software (NIH, USA) and normalised to GAPDH expression. All statistical analyses were performed using GraphPad Prism 8.0 software (GraphPad Software, San Diego, CA, USA). *p* < 0.05 was considered statistically significant. In figures, statistical significance is indicated as follows: *p < 0.05, **p < 0.01, ***p < 0.001.

## 3 Results

### 3.1 TAB2 Activates NF‐κB Signalling Pathway in CC Cells

**FIGURE 1:**
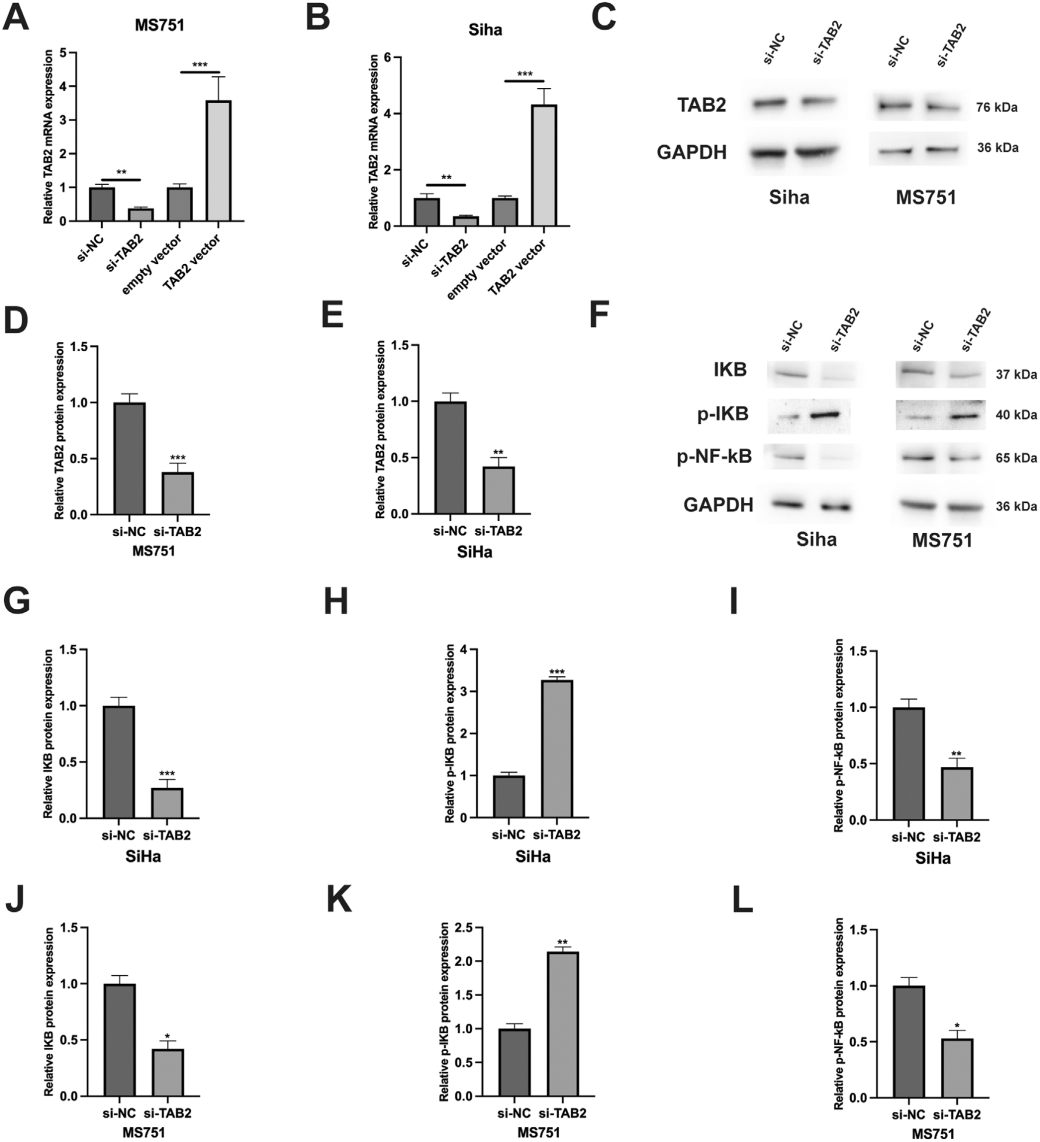
TAB2 regulates NF‐κB signalling pathway in cervical cancer cells. (A, B) qRT‐PCR analysis of TAB2 mRNA expression in Siha cells transfected with TAB2 overexpression vector or empty vector (2 μg/mL, 48 h). GAPDH served as internal control (*n* = 3 independent experiments). (C) Western blot analysis of TAB2 protein expression in Siha and MS751 cells transfected with si‐TAB2 or si‐NC (50 nM, 48 h). GAPDH served as loading control. (D, E) Quantification of TAB2 protein levels in Siha and MS751 cells using ImageJ software, normalised to GAPDH (*n* = 3 independent experiments). (F) Western blot analysis of IKB, p‐IKB and p‐NF‐κB expression in Siha and MS751 cells transfected with si‐TAB2 or si‐NC. (G–L) Quantification of IKB, p‐IKB and p‐NF‐κB protein levels in Siha and MS751 cells. Data are presented as mean ± SD from three independent experiments. Statistical analysis was performed using Student's *t*‐test. *p < 0.05, **p < 0.01, ***p < 0.001 versus control group.

Our previous findings indicated that TAB2 plays a crucial role in CC progression [10], prompting us to investigate the underlying molecular mechanisms. Given that TAB2 is known to function as an adapter protein in the NF‐κB signalling pathway through its interaction with TAK1 [11, 12], we hypothesised that TAB2 might regulate CC progression through the NF‐κB pathway. To investigate the role of TAB2 in NF‐κB signalling, we first manipulated TAB2 expression in CC cells. TAB2 overexpression significantly increased its mRNA levels in cells (Figure 1A,B), while siRNA‐mediated knockdown effectively reduced TAB2 protein expression in both Siha and MS751 cells (Figure 1C–E). We then examined the effect of TAB2 knockdown on NF‐κB pathway components. Western blot analysis showed that TAB2 silencing decreased IKB protein levels while increasing phosphorylated IKB (p‐IKB) levels in both Siha and MS751 cells (Figure 1F–H,J,K). Notably, phosphorylation of NF‐κB was significantly reduced following TAB2 knockdown in both cell lines (Figure 1I,L), suggesting that TAB2 is required for NF‐κB pathway activation in CC cells.

### 3.2 TAB2 Promotes Cancer Stemness Through NF‐κB Pathway

**FIGURE 2:**
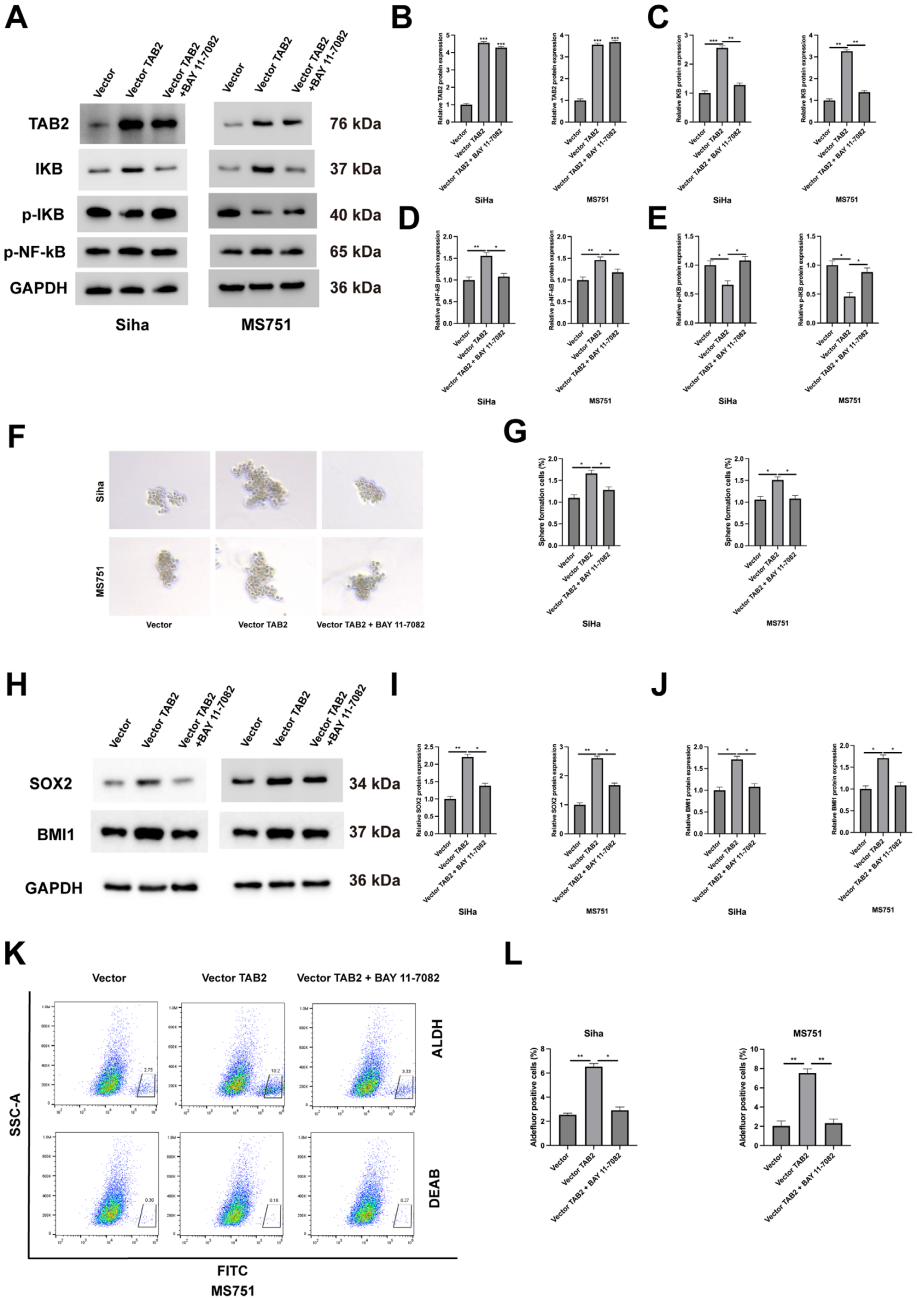
TAB2 promotes cancer stem cell properties through NF‐κB pathway activation. (A) Western blot analysis of TAB2, IKB, p‐IKB and p‐NF‐κB in Siha and MS751 cells transfected with empty vector, vector‐TAB2 (2 μg/mL) or vector‐TAB2 + BAY 11–7082 (5 μM, 24 h treatment). (B–E) Quantification of protein levels normalised to GAPDH (*n* = 3 independent experiments). (F) Representative images of sphere formation in Siha and MS751 cells with indicated treatments. Scale bar = 100 μm. (G) Quantification of sphere formation efficiency (spheres > 75 μm counted after 14 days, *n* = 3 independent experiments, 6 wells per condition). (H) Western blot analysis of stem cell markers SOX2 and BMI1. (I, J) Quantification of SOX2 and BMI1 protein levels. (K, L) Flow cytometry analysis of ALDH activity in MS751 cells and quantification (*n* = 3 independent experiments). DEAB served as negative control. Data are presented as mean ± SD. Statistical analysis was performed using one‐way ANOVA followed by Tukey's post hoc test. *p < 0.05, **p < 0.01, ***p < 0.001.

To investigate whether TAB2 regulates cancer stemness through NF‐κB signalling, we overexpressed TAB2 in Siha and MS751 cells with or without the NF‐κB inhibitor BAY 11–7082. Western blot analysis showed that TAB2 overexpression significantly increased TAB2, IKB and p‐NF‐κB protein levels, while reducing p‐IKB levels. However, these effects were largely reversed by BAY 11–7082 treatment (Figure 2A–E). Functionally, TAB2 overexpression markedly enhanced the sphere formation capacity of both cell lines, whereas NF‐κB inhibition by BAY 11–7082 significantly impaired this effect (Figure 2F,G). Consistent with these findings, Western blot analysis revealed that TAB2 overexpression increased the expression of stem cell markers SOX2 and BMI1, which were also attenuated by BAY 11–7082 treatment (Figure 2H–J). Moreover, flow cytometry analysis demonstrated that TAB2‐induced ALDH activity, another indicator of cancer stemness, was effectively blocked by NF‐κB inhibition (Figure 2K,L). These results collectively suggest that TAB2 promotes cancer stem cell properties through activation of the NF‐κB pathway.

### 3.3 TAB2 Promotes CC Cell Growth and Migration Through NF‐κB Pathway

**FIGURE 3:**
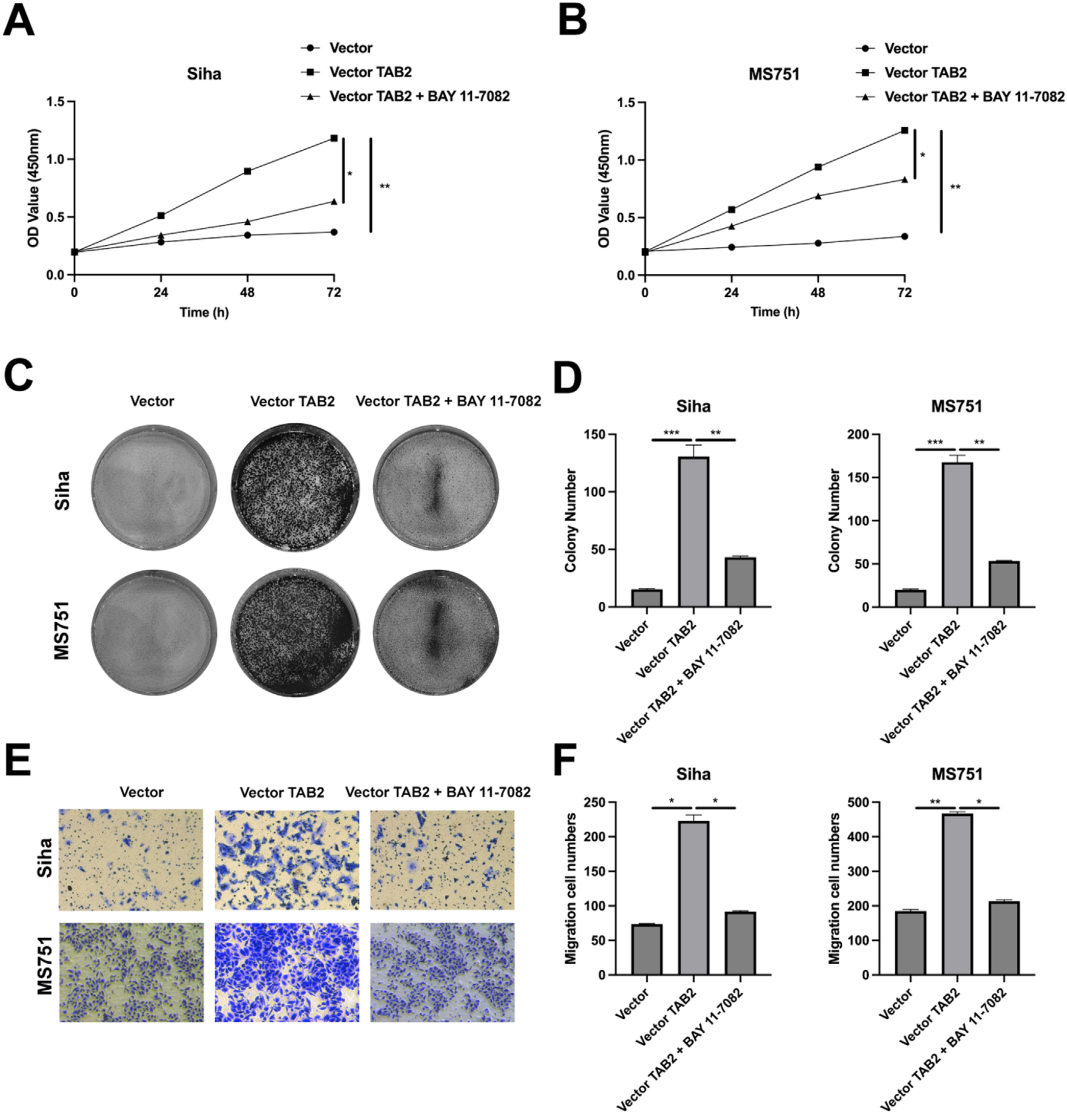
TAB2 promotes cervical cancer cell growth and migration through NF‐κB pathway. (A, B) Cell proliferation measured by CCK‐8 assay in Siha and MS751 cells transfected with empty vector, vector‐TAB2 (2 μg/mL) or vector‐TAB2 + BAY 11–7082 (5 μM). Absorbance was measured at 0, 24, 48 and 72 h (*n* = 3 independent experiments, 5 wells per condition). (C) Representative images of colony formation assay after 14 days. (D) Quantification of colony numbers (> 50 cells/colony, *n* = 3 independent experiments). (E) Representative images of Transwell migration assay after 24 h. Scale bar = 100 μm. (F) Quantification of migrated cells counted from five random fields per well (*n* = 3 independent experiments). Data are presented as mean ± SD. Statistical analysis was performed using two‐way ANOVA followed by Bonferroni's post hoc test for proliferation assays and one‐way ANOVA for colony formation and migration assays. *p < 0.05, **p < 0.01, ***p < 0.001.

We next investigated the functional role of TAB2 in CC cell growth and migration. Using CCK‐8 assays, we found that TAB2 overexpression significantly enhanced the proliferation of both Siha and MS751 cells over a 72‐h period compared to vector control. However, when cells were treated with the NF‐κB inhibitor BAY 11–7082, the TAB2‐induced proliferation was substantially suppressed (Figure 3A,B). To further confirm these findings, we performed colony formation assays. TAB2 overexpression markedly increased colony numbers in both cell lines, with approximately 6‐fold and 8‐fold increases in Siha and MS751 cells, respectively. This enhanced colony‐forming ability was significantly attenuated by BAY 11–7082 treatment (Figure 3C,D). We then examined the effect of TAB2 on cell migration using Transwell assays. Consistent with the proliferation results, TAB2 overexpression significantly enhanced the migratory capacity of both Siha and MS751 cells. The number of migrated cells increased by approximately 3‐fold in Siha cells and 2.5‐fold in MS751 cells compared to control groups. Notably, inhibition of NF‐κB signalling by BAY 11–7082 effectively reversed the TAB2‐induced migration (Figure 3E,F). These results collectively demonstrate that TAB2 promotes CC cell growth and migration through activation of the NF‐κB pathway.

### 3.4 TAB2 Promotes Immune Escape Through NF‐κB‐Dependent PD‐L1 Regulation

**FIGURE 4:**
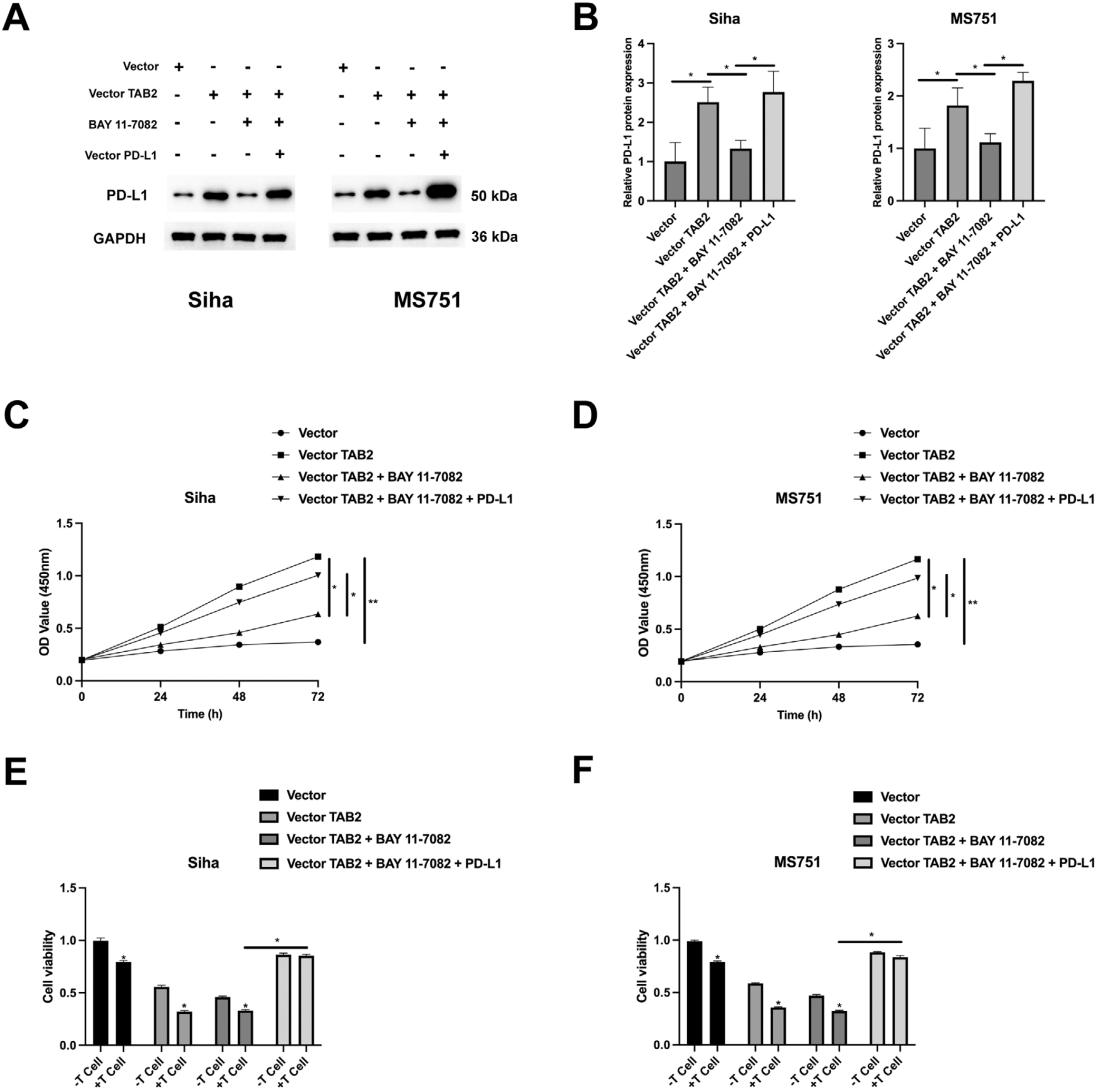
TAB2 promotes immune escape through NF‐κB‐dependent PD‐L1 regulation. (A) Western blot analysis of PD‐L1 protein expression in Siha and MS751 cells under different treatment conditions: Control vector, TAB2 overexpression vector (2 μg/mL), BAY 11–7082 (5 μM, 24 h) and PD‐L1 overexpression vector (2 μg/mL). GAPDH serves as loading control. (B) Quantification of relative PD‐L1 protein expression normalised to GAPDH (*n* = 3 independent experiments). (C, D) Cell proliferation curves measured by CCK‐8 assay (OD450nm) over 72 h showing the effects of different treatments in (C) Siha and (D) MS751 cells (*n* = 3 independent experiments, 5 wells per condition). (E, F) Cell viability assays showing the effects of T cell co‐culture (T cell: Cancer cell ratios = 0:1, 5:1, 10:1, 24 h co‐culture) on (E) Siha and (F) MS751 cells under different treatment conditions. Data are presented as mean ± SD. Statistical analysis was performed using one‐way ANOVA followed by Tukey's post hoc test. *p < 0.05, **p < 0.01.

Given the established role of NF‐κB signalling in immune regulation [13, 14, 15, 16], we investigated whether TAB2 might influence immune escape in CC cells. TAB2 regulates PD‐L1 expression through NF‐κB signalling in CC cells. Western blot analysis revealed that TAB2 overexpression significantly upregulated PD‐L1 protein levels by approximately 2.5‐fold in Siha cells and 1.8‐fold in MS751 cells compared to vector controls (Figure 4A,B). Treatment with the NF‐κB inhibitor BAY 11–7082 reversed this TAB2‐induced PD‐L1 expression to baseline levels. Notably, exogenous PD‐L1 expression effectively restored PD‐L1 levels in BAY 11‐7082‐treated cells, confirming the specificity of this regulation. To evaluate the functional significance of this TAB2‐NF‐κB‐PD‐L1 axis, we assessed cancer cell sensitivity to T cell‐mediated killing. Cell viability assays demonstrated that TAB2 overexpression significantly protected both Siha and MS751 cells from T cell‐mediated cytotoxicity (Figure 4C–F). This protective effect was abolished by BAY 11–7082 treatment, indicating the requirement of NF‐κB activation. Importantly, restoring PD‐L1 expression rescued the protective effect against T cell‐mediated killing in BAY 11‐7082‐treated cells.

### 3.5 TAB2 Mediates Chemoresistance in CC Cells

**FIGURE 5:**
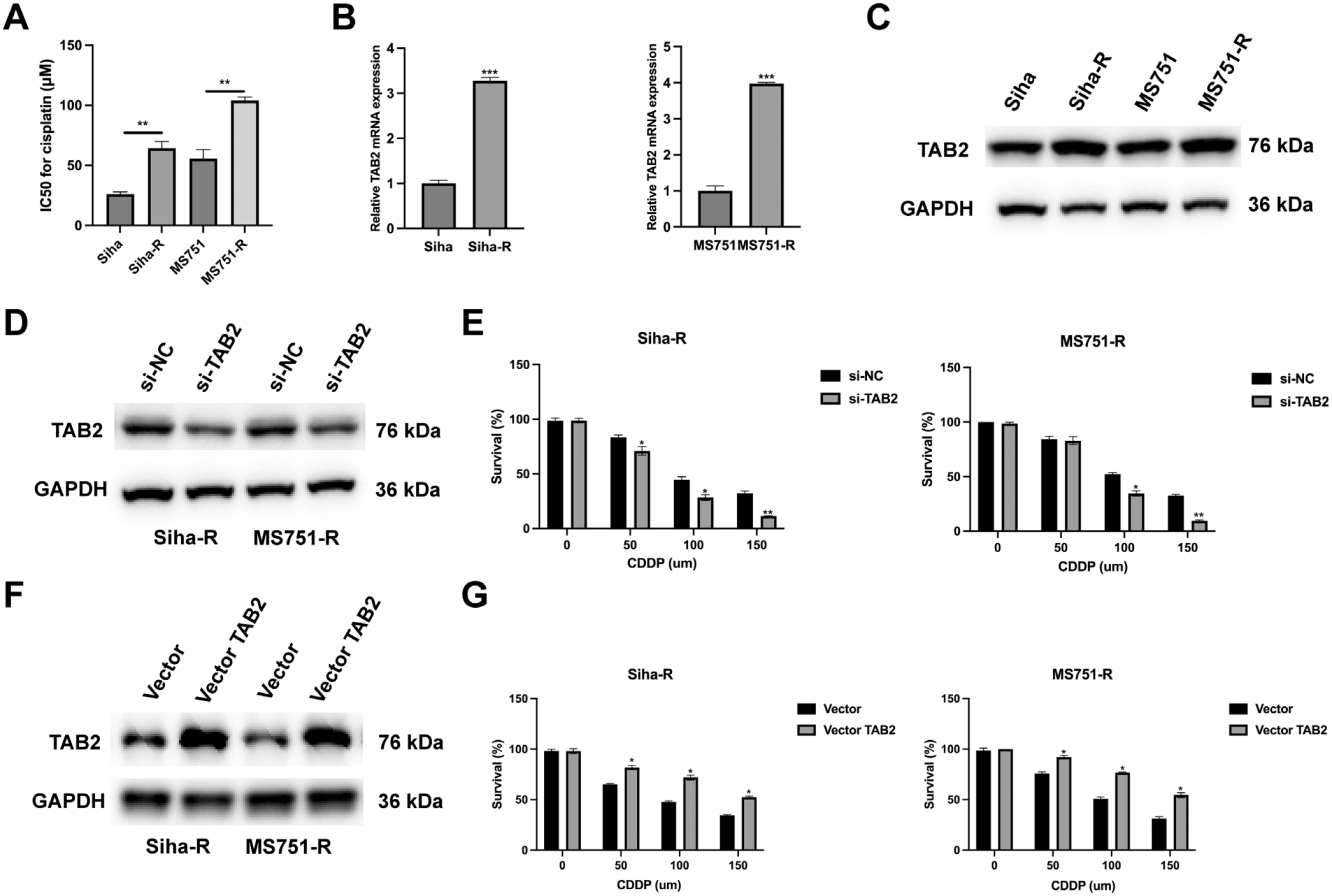
TAB2 promotes cisplatin resistance in cervical cancer cells. (A) IC50 values for cisplatin in parental and resistant cervical cancer cells treated with various concentrations (0–150 μM) for 48 h (*n* = 3 independent experiments). (B) qRT‐PCR analysis of TAB2 mRNA expression in Siha/Siha‐R and MS751/MS751‐R cells. GAPDH served as internal control (*n* = 3 independent experiments). (C) Western blot analysis of TAB2 protein levels in parental and resistant cells. (D) Western blot confirmation of TAB2 knockdown in resistant cells (si‐TAB2, 50 nM, 48 h). (E) Cell survival analysis of resistant cells transfected with si‐NC or si‐TAB2 under different concentrations of cisplatin treatment (48 h). (F) Western blot confirmation of TAB2 overexpression in resistant cells (vector‐TAB2, 2 μg/mL, 48 h). (G) Cell survival analysis of resistant cells transfected with vector or vector‐TAB2 under different concentrations of cisplatin treatment. Data are presented as mean ± SD from three independent experiments. Statistical analysis was performed using Student's *t*‐test for expression analysis and two‐way ANOVA followed by Bonferroni's post hoc test for survival analyses. *p < 0.05, **p < 0.01, ***p < 0.001.

Our previous results demonstrated that TAB2 promotes cancer stemness through NF‐κB pathway activation, as evidenced by enhanced sphere formation capacity and increased expression of stem cell markers SOX2 and BMI1. Given that CSCs are widely recognised as key contributors to chemoresistance, and our findings showed TAB2's crucial role in maintaining stemness properties, we hypothesised that TAB2 might be involved in chemoresistance in CC. To test this hypothesis, we first established cisplatin‐resistant cell lines (Siha‐R and MS751‐R) through gradual exposure to increasing concentrations of cisplatin. IC50 analysis confirmed significantly higher cisplatin tolerance in resistant cells compared to parental cells (Figure 5A). Importantly, both resistant cell lines showed markedly elevated TAB2 expression at mRNA and protein levels (Figure 5B,C). To determine whether TAB2 functionally contributes to cisplatin resistance, we performed TAB2 knockdown in resistant cells (Figure 5D). Cell survival assays revealed that TAB2 silencing significantly sensitised resistant cells to cisplatin treatment (Figure 5E). Conversely, TAB2 overexpression (Figure 5F) enhanced cell survival under cisplatin treatment in both resistant cell lines (Figure 5G). These findings establish TAB2 as a critical mediator of cisplatin resistance in CC cells.

### 3.6 TAB2 Promotes Chemoresistance Through NF‐κB Pathway Activation in Resistant Cells

**FIGURE 6:**
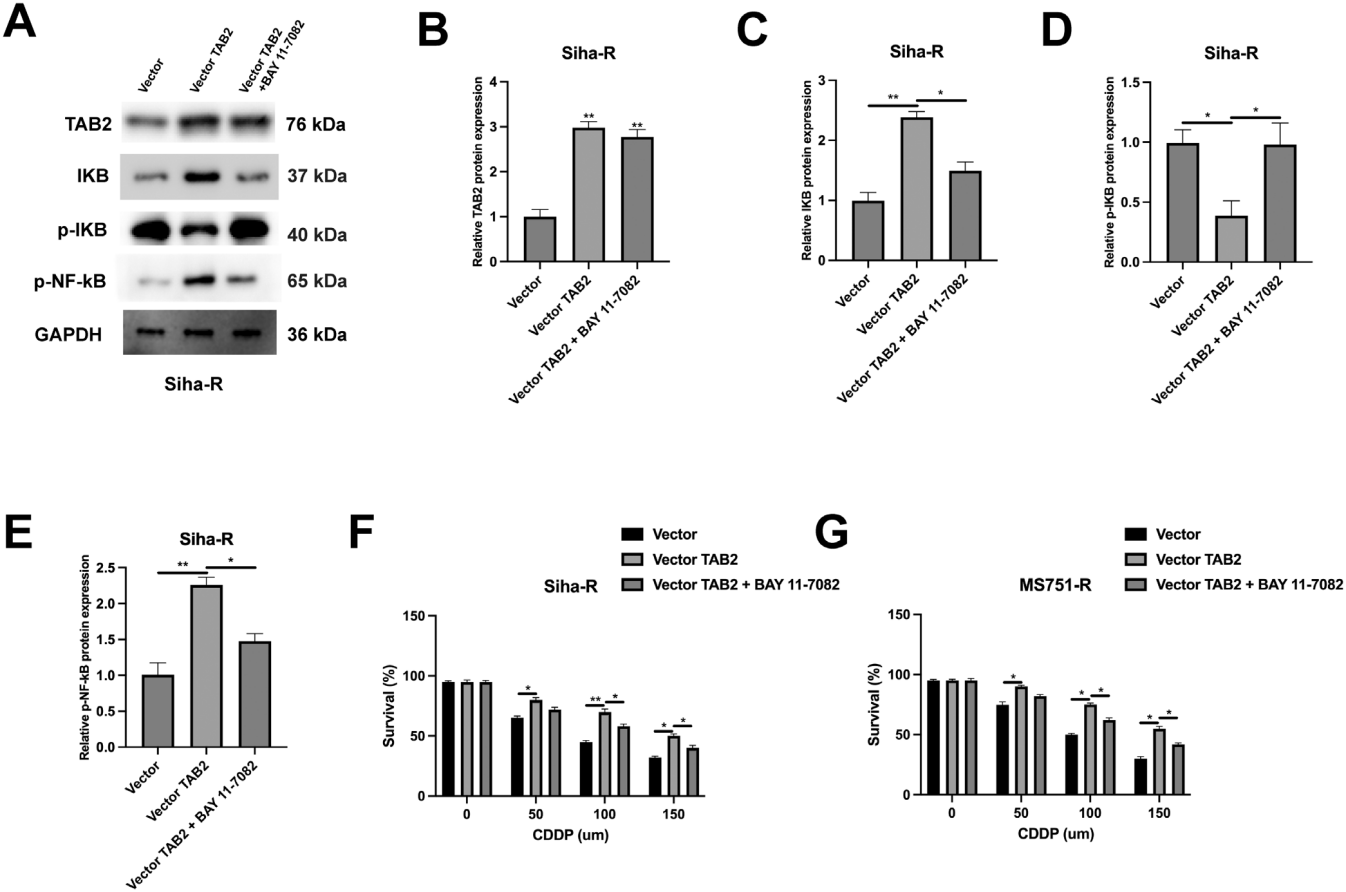
TAB2 promotes chemoresistance through NF‐κB pathway activation. (A) Western blot analysis of TAB2, IKB, p‐IKB and p‐NF‐κB in Siha‐R cells transfected with empty vector or vector‐TAB2 (2 μg/mL), with or without BAY 11–7082 treatment (5 μM, 24 h). (B–E) Quantification of relative protein levels normalised to GAPDH from Western blot analysis (*n* = 3 independent experiments). (F, G) Cell survival analysis of Siha‐R and MS751‐R cells with indicated treatments under different concentrations of cisplatin (0–150 μM, 48 h treatment). Data are presented as mean ± SD from three independent experiments. Statistical analysis was performed using one‐way ANOVA followed by Tukey's post hoc test for Western blot quantification and two‐way ANOVA followed by Bonferroni's post hoc test for survival analyses. *p < 0.05, **p < 0.01.

Having established TAB2's involvement in cisplatin resistance, we next investigated whether this effect is mediated through NF‐κB signalling in resistant cells. Western blot analysis revealed that TAB2 overexpression in Siha‐R cells increased the levels of IKB and phosphorylated NF‐κB, while BAY 11–7082 treatment reversed these effects (Figure 6A–E). To determine whether NF‐κB pathway activation is required for TAB2‐mediated chemoresistance, we examined cell survival under cisplatin treatment. TAB2 overexpression significantly enhanced cell survival in both Siha‐R and MS751‐R cells across various cisplatin concentrations. However, this protective effect was largely abolished by BAY 11–7082 treatment (Figure 6F,G), suggesting that TAB2 promotes cisplatin resistance through NF‐κB pathway activation.

## 4 Discussion

In this study, we have uncovered several critical roles of TAB2 in CC progression through NF‐κB pathway regulation. Our findings demonstrate that TAB2 modulates multiple aggressive cancer phenotypes, including enhanced cell proliferation, increased migration capacity and pronounced stemness characteristics, all through NF‐κB signalling activation. Notably, we identified TAB2 as a crucial mediator of both immune escape and chemoresistance mechanisms, as evidenced by its regulation of PD‐L1 expression and elevated levels in cisplatin‐resistant cells. These findings establish TAB2 as a multifaceted regulator of CC progression and highlight its potential as a therapeutic target.

TAB2 serves as a critical adapter protein that mediates NF‐κB signalling pathway activation through its interaction with TAK1. In this study, we demonstrated that TAB2 functions as an essential upstream regulator of NF‐κB activation in CC cells, where knockdown of TAB2 significantly decreased phosphorylation of IκB and NF‐κB. This molecular mechanism aligns with previous findings in bronchial epithelial cell inflammation, where TAB2 connects transforming growth factor beta‐activated kinase 1 (TAK1) and TNF receptor‐associated factor 6 (TRAF6), enabling TAK1 to activate IKKs which promote NF‐κB nuclear translocation [17]. Similarly, in COPD tissue, the TAB2/NF‐κB signalling axis has been shown to be crucial for inflammatory responses, with TAB2 functioning upstream of the MAPK and NF‐κB signalling pathways [12]. The significance of TAB2‐mediated NF‐κB activation in CC is highlighted by its central role in promoting multiple aggressive phenotypes—our data revealed that TAB2 not only drives inflammatory responses but also regulates immune escape through PD‐L1 expression and contributes to chemoresistance development. These findings are particularly important as they demonstrate TAB2's position as a central regulator where it can simultaneously modulate multiple hallmarks of cancer progression through NF‐κB pathway activation in CC cells.

Our research highlights a novel mechanism wherein TAB2 regulates PD‐L1 expression in CC cells via the NF‐κB signalling pathway. Specifically, TAB2 overexpression significantly upregulates PD‐L1 protein levels, an effect that is effectively blocked by the NF‐κB inhibitor BAY 11–7082, confirming the NF‐κB dependency. Functionally, TAB2 overexpression enhances the protection of CC cells against T cell‐mediated cytotoxicity, which can be abolished by NF‐κB inhibition. This firmly establishes TAB2 as a central regulator simultaneously modulating chemoresistance and immune escape through NF‐κB pathway activation. Furthermore, TAB2's influence extends beyond direct PD‐L1 regulation, potentially affecting the tumour immune microenvironment by impacting inflammatory cytokine production and immune cell infiltration. This is supported by other research demonstrating the role of NF‐κB activation in upregulating PD‐L1 in various cancers, and the involvement of miRNAs like miR‐149‐5p in regulating inflammation and immune responses via TAB2 [17]. Moreover, studies on dry eye disease (DED) show that hucMSC‐EVs can target the IRAK1/TAB2/NF‐κB pathway with miRNAs, inhibiting inflammation and costunolide reduces myocardial inflammation by inhibiting the TAK1/TAB2 complex [18]. Given that NF‐κB activation promotes tumour progression, including cell proliferation, angiogenesis and metastasis, and that inhibiting NF‐κB can lead to tumour regression, our findings suggest that targeting TAB2 could enhance immunotherapy efficacy by reducing PD‐L1 expression and modulating the immunosuppressive microenvironment, making it a promising target, especially in combination with immune checkpoint inhibitors for CC treatment.

The nuclear factor‐kappa B (NF‐κB) signalling pathway plays a significant role in promoting cancer cell stemness and chemoresistance [19, 20, 21, 22, 23, 24, 25]. Cancer stem cells, which possess the ability to self‐renew and differentiate into various cell types, are often implicated in tumour initiation, metastasis and recurrence, as well as resistance to therapies. Activation of the NF‐κB pathway in cancer cells can lead to the expression of genes that are involved in maintaining stemness properties, such as those related to cell survival, proliferation and epithelial‐mesenchymal transition (EMT) [26]. Specifically, the NF‐κB pathway can contribute to the formation of tumour‐initiating cells by increasing Wnt signalling, which can cause non‐stem cells to dedifferentiate [27]. Furthermore, NF‐κB can promote the expression of stemness‐related factors like Gal‐3, which has been shown to increase signals related to stemness and cell motility. In the context of chemoresistance, the NF‐κB pathway can activate genes involved in drug efflux, detoxification and DNA repair, thus protecting cancer cells from the cytotoxic effects of chemotherapeutic agents. The NF‐κB pathway can also promote an inflammatory microenvironment that further contributes to drug resistance by altering local metabolism and making the immune system ineffective. For example, the activation of NF‐κB can lead to the production of cytokines and chemokines that promote cancer cell survival and proliferation while simultaneously suppressing the immune response. Overall, the NF‐κB pathway contributes to cancer cell stemness and chemoresistance by inducing the expression of genes that promote cell survival, proliferation, EMT and drug resistance while also suppressing the immune response [28].

Based on the established role of NF‐κB signalling in promoting cancer cell stemness and chemoresistance, and our findings that TAB2 activates the NF‐κB pathway in CC cells, we hypothesised that TAB2 might regulate chemoresistance. Previous studies have shown that NF‐κB activation maintains stemness properties through regulating cell survival, proliferation and EMT, while also contributing to drug resistance by activating genes involved in drug efflux, detoxification and DNA repair. Indeed, we found TAB2 was significantly upregulated in cisplatin‐resistant CC cells, and its expression level correlated with drug resistance. Functional studies demonstrated that TAB2 knockdown sensitised resistant cells to cisplatin treatment, while TAB2 overexpression enhanced drug resistance. Mechanistically, inhibition of NF‐κB signalling by BAY 11–7082 reversed TAB2‐induced chemoresistance, confirming that TAB2 promotes drug resistance through the NF‐κB pathway. These findings establish TAB2 as a crucial mediator of chemoresistance in CC and suggest its potential as a therapeutic target.

Our study demonstrates that TAB2 coordinates multiple aspects of cancer progression through NF‐κB signalling. The regulation of PD‐L1 expression by TAB2 occurs specifically through NF‐κB activation, as evidenced by the reversal of TAB2's effects with BAY 11–7082 treatment. Similarly, the increased stemness properties and chemoresistance in TAB2‐overexpressing cells share this NF‐κB dependency, suggesting a common mechanism. The consistent ability of NF‐κB inhibition to block TAB2's effects across all phenotypes—from cell growth to immune escape—indicates that this pathway serves as the primary mediator of TAB2's functions in CC progression.

Several questions remain to be addressed in future studies. First, the detailed molecular mechanisms by which TAB2 activates NF‐κB signalling in CC cells need further investigation. Second, the potential role of TAB2 in regulating other cancer‐related pathways warrants exploration. Additionally, clinical studies are needed to validate the correlation between TAB2 expression and patient outcomes, as well as to develop effective TAB2‐targeting strategies. Understanding these aspects will be crucial for translating our findings into therapeutic applications.

Our findings reveal several promising therapeutic implications for CC treatment. First, since TAB2 overexpression contributes to both chemoresistance and immune escape, measuring TAB2 levels could potentially help predict which patients might be resistant to standard treatments. Second, our discovery that TAB2 works through the NF‐κB pathway suggests that existing NF‐κB inhibitors might be effective in treating resistant tumours, particularly when combined with standard chemotherapy. Finally, because we found that TAB2 regulates PD‐L1 expression, targeting TAB2 could potentially improve the effectiveness of immunotherapy. Together, these findings suggest that TAB2‐targeted treatments, either alone or in combination with existing therapies, could offer new options for CC patients who do not respond well to current treatments.

## Author Contributions

**Man Wu:** data curation (equal), investigation (equal), software (equal), validation (equal), visualization (equal). **Yingying Zhang:** data curation (equal), investigation (equal), software (equal), visualization (equal). **Xuanhui Wang:** data curation (equal), methodology (equal), project administration (equal), resources (equal), visualization (equal). **Yijia Zhou:** conceptualization (equal), investigation (equal), methodology (equal), project administration (equal), resources (equal), writing – original draft (equal), writing – review and editing (equal).

## Conflicts of Interest

The authors declare no conflicts of interest.

## Data Availability

Data available on request from the authors.
